# Expression of Fungal Cutinase and Swollenin in Tobacco Chloroplasts Reveals Novel Enzyme Functions and/or Substrates

**DOI:** 10.1371/journal.pone.0057187

**Published:** 2013-02-25

**Authors:** Dheeraj Verma, Shuangxia Jin, Anderson Kanagaraj, Nameirakpam D. Singh, Jaiyanth Daniel, Pappachan E. Kolattukudy, Michael Miller, Henry Daniell

**Affiliations:** 1 Burnett School of Biomedical Sciences, College of Medicine, University of Central Florida, Orlando, Florida, United States of America; 2 Research Instrumentation Facility, Auburn University, Auburn, Alabama, United States of America; Cinvestav, Mexico

## Abstract

In order to produce low-cost biomass hydrolyzing enzymes, transplastomic lines were generated that expressed cutinase or swollenin within chloroplasts. While swollenin expressing plants were homoplasmic, cutinase transplastomic lines remained heteroplasmic. Both transplastomic lines showed interesting modifications in their phenotype, chloroplast structure, and functions. Ultrastructural analysis of chloroplasts from cutinase- and swollenin-expressing plants did not show typical lens shape and granal stacks. But, their thylakoid membranes showed unique scroll like structures and chloroplast envelope displayed protrusions, stretching into the cytoplasm. Unusual honeycomb structures typically observed in etioplasts were observed in mature chloroplasts expressing swollenin. Treatment of cotton fiber with chloroplast-derived swollenin showed enlarged segments and the intertwined inner fibers were irreversibly unwound and fully opened up due to expansin activity of swollenin, causing disruption of hydrogen bonds in cellulose fibers. Cutinase transplastomic plants showed esterase and lipase activity, while swollenin transplastomic lines lacked such enzyme activities. Higher plants contain two major galactolipids, monogalactosyldiacylglycerol (MGDG) and digalactosyldiacylglycerol (DGDG), in their chloroplast thylakoid membranes that play distinct roles in their structural organization. Surprisingly, purified cutinase effectively hydrolyzed DGDG to MGDG, showing alpha galactosidase activity. Such hydrolysis resulted in unstacking of granal thylakoids in chloroplasts and other structural changes. These results demonstrate DGDG as novel substrate and function for cutinase. Both MGDG and DGDG were reduced up to 47.7% and 39.7% in cutinase and 68.5% and 67.5% in swollenin expressing plants. Novel properties and functions of both enzymes reported here for the first time should lead to better understanding and enhanced biomass hydrolysis.

## Introduction

Lignocellulosic biomass is a valuable bioethanol production source for substitution of established fuel supply. The translation of lignocellulosic biomass into bioethanol depends on release of sugars by breakdown of complex assembly of cellulosic and hemicellulosic units in biomass. Hydrolysis of complex biomass requires chemical or physical pretreatment and large amount of distinct group of enzymes including cellulases, hemicellulases and accessory enzymes [1], [2]. Often, pretreatments are expensive, eco-destructive and inhibit downstream processing of sugars into fuel. In addition, current production system of enzymes through fermentation is costly and incapable of producing enzymes in bulk quantities [1], [2]. The chloroplast-derived enzyme cocktails for hydrolysis of lignocellulosic biomass address the concerns of high cost and poor creation facility [1], [3], [4]. Chloroplasts carry out oxygenic photosynthesis to produce food as well as oxygen and sustain life on earth. Photosynthetic energy transduction and the generation of plant biomass rely on the thylakoid membranes inside chloroplasts. Well-developed intricate and expansive thylakoid membrane system is vital for the photosynthetic reactions in higher plants. The thylakoid membrane of chloroplast is predominantly made of galactolipids. Monogalactosyldiacylglycerol (MGDG) and digalactosyldiacylglycerol (DGDG) constitute more than 80% of total galactolipids in thylakoid membranes. Therefore, MGDG and DGDG are important for all oxygenic photosynthetic organisms. These two galactolipids of thylakoid membrane are most abundant lipids in the biosphere [5], [6]. Thylakoid membrane also has other lipids like sulfoquinovosyldiacylglycerol (SQDG) and phosphatidylglycerol (PG). MGDG on its own cannot make lamellar membranes whereas along with DGDG, proteins and xanthophylls, lamellar membranes are formed with intrinsic curvature stress [7]. Hydrogen bond formation between the galactolipid head groups of MGDG and DGDG is important for these lipids to associate with photosystem I (PSI) and photosystem II (PSII). Thus, an optimal lipid-protein ratio is maintained which is crucial for development, maturation process, fluidity and integrity of thylakoid membrane [8]–[11].

MGDG and DGDG have two extremely unsaturated fatty acyl chains and differ from the majority of natural lipids having one saturated and one unsaturated chain. The head group of DGDG is relatively large and is anticipated to be cylindrical and form bilayer, whereas the shape of MGDG is conical and pack into the curved hexagonal H_II_ structure [12]–[14]. Both MGDG and DGDG are linked to light harvesting complex (LHC II) and have distinct roles in structural organization of the thylakoid membranes. LHC II form trimers and associate with DGDG molecules at the trimer-trimer interface facilitating formation of crystalline arrays through hydrogen bonds and hydrophobic interactions [14], [15]. The elimination of DGDG using mild detergent treatment or anion exchange chromatography has been found to cause the LHC II to lose the potential of forming two- or three-dimensional crystals [15]. In stacked granal thylakoids, electrostatic interactions between super complexes of PSII and LHCII occasionally form ordered arrays containing two dimensional crystals of super complexes in each membrane on both sides of the stromal gap [16]. In unstacked membranes, the ordered arrays are lost [16]. Several multisubunit protein complexes like PSI, PSII, LHC-I, LHC-II, cytochrome b_6_/f complex and ATP synthase are embedded in thylakoid membranes [17], [18]. Therefore, disruption of the protein complexes and disorganization of thylakoid membranes could result in adverse phenotype in plants.

Swollenin is an expansin-like protein from *Trichoderma reesei* with an N-terminal cellulose binding domain (CBD) connected to the expansin-like domain with a linker region [19]. Similar to plant expansins, swollenin expressed in yeast displayed weakening of tensile strength of filter paper. It also disrupted cotton fibers framework displaying swollen areas without release of reducing sugars. Expansins have been suggested to loosen the meshwork by weakening hydrogen bonds between cellulose fibers or microfibrils, without any hydrolytic activity on cellulose molecules, causing smooth movement of cellulose fibers and expansion of the cell wall [20], [21]. This results in separation of cellulose microfibrils and cell wall extension and enlargement in growing cells [22], [23]. The plant expansins are encoded by muti-gene families and are grouped into four families: α, β, expansin-like A and expansin-like B. The α and β- expansins tightly bind to different regions of cell wall polymers and cause cell wall extension and enlargement, whereas mode of action of expansin-like A and B proteins are not well understood [22]. Transgenic plants developed via nuclear expression of expansin genes isolated from different plants species caused various physiological changes including cell expansion in primary tissues, secondary tissues, sensitivity to salt stress, root elongation, fruit softening, leaf development and growth pattern [24]–[30]. In another report, the loosening of plant cell structure was attributed to proteolytic activity of the *Phleum pratense* expansin by cleaving cell wall proteins holding together cellulose fibers instead of disrupting hydrogen bonding between fibers [31]. Expansins also influence many physiological processes where cell enlargement occurs including pollen tube invasion of the stigma, fruit ripening and softening, organ abscission, leaf organogenesis and seed germination [32]. Manipulation of swollenin expression levels in *Trichoderma* mutants confirmed role of swollenin gene in plant root colonization [33]. Expansins are also involved in syncytium formation in *Arabidopsis thaliana* roots upon nematode infection [34]. In a recent report, a gene coding for a swollenin-like protein (AfSwo1) from *Aspergillus fumigatus* showed weak endoglucanase activity [35].

Cutinases are inducible extracellular enzymes secreted by microorganisms to break down plant cell walls. Cutinases from a number of fungi have been purified and described [36]–[43]. In contrast to fungi, very few bacteria have been reported to produce cutinase [44], [45]. Recently, two cutin-induced cutinases were cloned, expressed and purified from *Thermobifida fusca* and their biochemical properties were investigated [46]. The primary function of cutinase is to hydrolyze cutin [47]–[49]. Arabidopsis plants overexpressing cutinase from *Fusarium solani* f sp *pisi* revealed modifications in ultrastructure of the cuticle and developmental abnormalities including postgenital organ fusions and morphological changes in epidermal layer [50].

Because, cutinase and swollenin are important accessory enzymes for enhancing the hydrolysis of lignocellulosic biomass and cutin doesn’t exist within chloroplasts, expression of these enzymes in tobacco chloroplasts was investigated. Surprisingly, we observed a new substrate (DGDG) and functions for both cutinase and swollenin. The impact of cutinase and swollenin expression on plant phenotype and chloroplast ultrastructure was also investigated.

## Materials and Methods

### Vector Construction and Regeneration of Transplastomic Plants

Full length *swo*I (AJ245918) coding sequence was assembled using overlapping primers of various exons (Exon 1–5′ GAA TTC CAT ATG GCT GGT AAG CTT ATC CTC GTG GC 3′ & 5′ CCA CAT TGG CCA AAT AAT GCT GCG CAA TTC TGC TG 3′; Exon 2–5′ CGC AGC ATT ATT TGG CCA ATG TGG AGG CAT AGG GT 3′ & 5′ GGG GTT TCC GCC GGT TGA CGC AAG GCA CTG GGA G 3′; Exon 3–5′ CTT GCG TCA ACC GGC GGA AAC CCC CCA AAC GGA 3′ & 5′ GTG GGT TGA TCT ACT GTA GTG CCA GCT CTC GTT GC 3′; Exon 4–5′ TGG CAC TAC AGT AGA TCA ACC CAC TTT GGC CTA ACG A 3′ & 5′ CGA GGA CCA GAA TTG GGT GTA GTA ATC GCC GTT TGG 3′; Exon 5–5′ TAC TAC ACC CAA TTC TGG TCC TCG TTG CCA GGA 3′ & 5′ CGC CCG GAC CAC AGC ACC ACT TGG AGT TCG CGC T 3′; Exon 6–5′ CCA AGT GGT GCT GTG GTC CGG GCG CCG ATC A 3′ & 5′ ATG CTC TAG ATC AAT TCT GGC TAA ACT GCA CAC C 3′) and *Trichoderma reesei* genomic DNA by a PCR-based method [51] and *cutinase* gene was amplified using gene-specific primers (5′ TGA ATT CCA TAT GCA TCA TCA TCA TCA TCA CAA ATT CTT CGC TCT CAC CAC ACT TC 3′ & 5′ TGC TCT AGA TCA AGC AGA ACC ACG GAC AGC C 3′) from recombinant clone of *Fusarium solani* [48] as described earlier [1]. The pCR Blunt II Topo vector (Invitrogen) was used to subclone amplified PCR products. After sequencing (Genewiz), each gene was ligated into the pLD vector [52], [53] to create the tobacco chloroplast expression vector. Tobacco (*Nicotiana tabacum* var. Petite Havana) seeds were germinated under sterile conditions on half-strength Murashige and Skoog (MS) agar solidified medium supplemented with 30 g/l sucrose and without hormones. Sterile young leaves were transformed via particle bombardment using pLD-cut or pLD-swo1 plasmid DNA coated on gold particles. Transplastomic plants were selected and raised as described earlier [54], [55].

### Confirmation of Transgene Integration by PCR and Southern Blot

Plant total DNA was extracted from the spectinomycin resistant and untransformed shoots using Qiagen DNeasy plant mini kit according to manufacturer’s guidelines. PCR was carried out to verify precise integration of transgene into the chloroplast’s genome inverted repeat region exploiting two primer pair sets as described previously [54], [55]. After PCR confirmation, small sections of leaves were made and kept on regeneration medium with spectinomycin for second round of selection. Regenerated shoots were subjected to third round of selection for achieving homoplasmy by transferring shoots to ½ MSO medium (growth hormone and vitamin free ½ MS salts plus 2% sucrose) supplemented with spectinomycin.

Southern blot study was done in accordance with published lab protocols [54], [55]. Concisely, leaves were harvested from shoots growing in third round of selection and total genomic DNA was extracted. One to two micrograms of DNA was treated with *Sma*I restriction enzyme and fractionated using agarose gel (0.8%) electrophoresis followed by transfer to a nylon membrane. The pUC-Ct vector DNA was cut with *Bam*HI and *Bgl*II to release a 0.81 kb probe [1]. The digested product was ^32^P α[dCTP] labeled and added to Stratagene QUICK-HYB hybridization solution. The nylon membrane was incubated in QUICK-HYB hybridization solution containing labeled probe for hybridization according to manufacturer’s protocol.

### Microscopic Analysis of Chloroplasts

For light microscopic observation of chloroplast phenotypes for transplastomic and untransformed lines, expanded leaves were sliced into little pieces and incubated for one and a half hour in 3.5% glutaraldehyde at room temperature under dark conditions. Afterwards, the pieces were incubated for 30 min in 0.1 M Na_2_EDTA (pH 9.0) at 50°C. The samples were kept at 4°C for few hrs before observing under a Leica inverted microscope outfitted with a Leica Camera (60×).

For transmission electron microscopy, small pieces of leaf (3–4 mm) dissected from transplastomic and untransformed plants were fixed in fixative (2.5% glutaraldehyde, 2% paraformaldehyde in 0.1 M phosphate buffer) at room temperature for 3–4 hrs. Tissues were rinsed four times (15 min each) with 0.1 M phosphate buffer and subsequently dipped in 2% Osmium tetroxide for 1–3 hours at room temperature under dark condition for secondary fixation. Following dehydration of the tissues with graded ethanol series (30% ethanol for 10 min; 50, 70, 80, 90, 95% ethanol for 20 min each; 3 times with 100% ethanol for 30 min), samples were treated twice (15 min each) with 100% propylene oxide. Prior to final embedding (60°C for 24 hrs) in EMbed812, dehydrated samples were serially soaked in 33.3%, 66.6% and 100% EMbed812 (in propylene oxide) for 3 hrs, overnight, and 1.5 hrs respectively. Samples were sectioned (70 nm) and dehydrated on 150-mesh copper grids. Uranyl acetate and lead citrate was used for post staining as explained earlier [56]. The grids were dried and samples were examined under Philips-Tecnai 12 transmission electron microscope.

### Enzyme Extraction from Transplastomic Leaves and *E.coli*

Enzyme extraction was carried out in the working concentration and pH of the appropriate reaction buffer (described below for cotton fiber swelling assay and esterase activity assay) after grinding the *in vitro* leaves to powder in liquid nitrogen. For *E. coli*, cells were collected at 4°C and sonicated using 30s pulse (four times) in suitable buffer on ice. The EDTA-free protease inhibitor cocktail (Roche) was added to all the buffers. Total soluble protein (TSP) was passed through a syringe filter (0.22 µm). Bradford assay was used to determine TSP concentration (mg/ml) employing spectrophotometric absorption at 595 nm absorption and deducting the turbidity of extracts at 700 nm as described previously [1].

### Cotton Fiber Swelling Assay

Cotton fibers of greenhouse grown plants of *Gossypium hirsutum* cv Coker 310FR [57] were mercerized with 25% NaOH at 4°C for 15 minutes according to Saloheimo et al protocol [19]. Mercerized cotton fibers were washed gently and thoroughly in distilled water. These fibers were suspended at a concentration of 250 mg/ml reaction volume containing the following buffers: 50 mM sodium acetate, pH5.0; 50 mM sodium phosphate buffer, pH 6.0; 100 mM Tris-HCl buffer, pH 7.0 and 7.5 at 25°C for 4 to 8 hours. Enzymes used were cpSwollenin (chloroplast derived), cpCutinase (chloroplast derived), *E. coli* Swollenin, *E. coli* Cutinase or untransformed plant total soluble protein in the range of 0.2 to 2.0 mg tsp per reaction. At the end of reaction, fibers were washed off in distilled water and observed under phase contrast light microscope (Olympus, 40X).

### Esterase and Lipase Activity Assay of Enzymes Expressed in Chloroplast

Tributyrin (1%) and para-nitrophenyl butyrate (pNPB, 0.01%) substrates were used for determining esterase activity following previously used protocol [50], [58] with following modifications. The cpSwollenin in the range of 0.2–2.0 mg tsp was assayed with 1% emulsified tributyrin in 2% agarose plate having punch hole diameter of 1.0 cm^2^ or 0.01% pNPB having 50 mM sodium acetate, pH5.0; 50 mM sodium phosphate buffer, pH 6.0; 100 mM Tris-HCl buffer, pH 7.0 and 7.5 and 8.0 with or without 0.03% Triton X-100 at 37°C or 50°C for 24 hours. Enzyme assay was carried out up to 30 minutes for pNPB substrate at 30°C. The cpCutinase tsp in the range of 50 µg to 2.0 mg was assayed in the buffer containing 100 mM Tris-HCl buffer, pH 7.0 and 7.5 and 8.0 with 0.03% Triton X-100 at 30°C, 15 minutes for pNPB and 30, 37 and 50°C, 24 hours for 1% emulsified tributyrin. One unit of cutinase enzyme activity is defined as the production of 1 µmol of p-nitrophenol per minute under the assay conditions [1].

### 14C Labeling of DGDG and Lipid Analysis

Healthy young expanding tobacco leaves from greenhouse grown plants were sliced under water from the leaf base to keep the transpiration stream undamaged. The cut leaf was placed upright in a beaker with cut end immersed in water and incubated for 2 hours in the dark. Leaves were then incubated in continuous light for 2 h with 250 µCi [1-^14^C] acetate sodium salt (American Radiolabeled Chemicals, St. Louis, MO) in 2 ml of water at 22–24°C so that [1-^14^C] acetate was taken up by transpiration. The transpired water was continuously replaced with 1 ml of water so that transpiration stream is uninterrupted. After 2 hours, the beaker was filled with 200 ml of water and leaf was incubated for 16 h in continuous light at 22–24°C. At the end of incubation period leaves were sliced into small pieces, transferred to an Erlenmeyer flask containing chloroform/methanol in a ratio of 2∶1 and stirred for 4 hours. The mixture was filtered through Whatman filter paper into separating funnel, acidified with 6N HCl and shaken vigorously. The mixture was then allowed to separate and organic layer was collected into a clean flask. The remaining water layer in separating funnel was treated twice with chloroform and the organic phase was collected. Organic solvents were evaporated from the flask using a vacuum rotary evaporator. The dried lipids were resuspended in small volume of chloroform/methanol (2∶1) and fractionated using thin layer chromatography in a solvent system comprised of chloroform/methanol/water in a ratio of 65∶25:2 (v/v/v). Radiolabeled lipids were visualized by autoradiography and in iodine vapor. Radiolabeled DGDG was identified by co-chromatography with authentic DGDG lipid standard (Sigma), scraped off, isolated from the silica material and associated radioactivity was determined by liquid scintillation counting.

Different leaves (Green, mosaic and white) from swollenin and cutinase expressing plants were harvested. Lipids were isolated and fractionated by thin layer chromatography as explained above. Lipid bands were visualized in iodine vapor and identification was done by comparison with available MGDG and DGDG standard lipids and with published diagrams for the same solvent system.

### Assay of Cutinase Activity on Radiolabeled DGDG

Cutinase (4 µg) from *Fusarium solani pisi* [59] was incubated for 2 h at 37°C in a reaction buffer containing 0.1 µCi ^14^C-DGDG, 100 mM Tris-HCl pH 8.0 and 0.025% Triton X-100. The 200 µl reaction was terminated by the addition of 50 µl 6N HCl and reaction products were extracted with 1 ml chloroform:methanol (2∶1, v/v) followed by two extractions with chloroform (1 ml) and were resolved on silica-TLC using chloroform:methanol:water (65∶25:2, v/v/v) as the solvent system. Cutinase was alternatively pre-incubated with paraoxon, an active serine-directed inhibitor, for 15 min prior to the assay. The radioactivity on the TLC plate was imaged by autoradiography at −80°C.

## Results

### Evaluation of Transgene Integration and Homoplasmy

**Figure 1:**
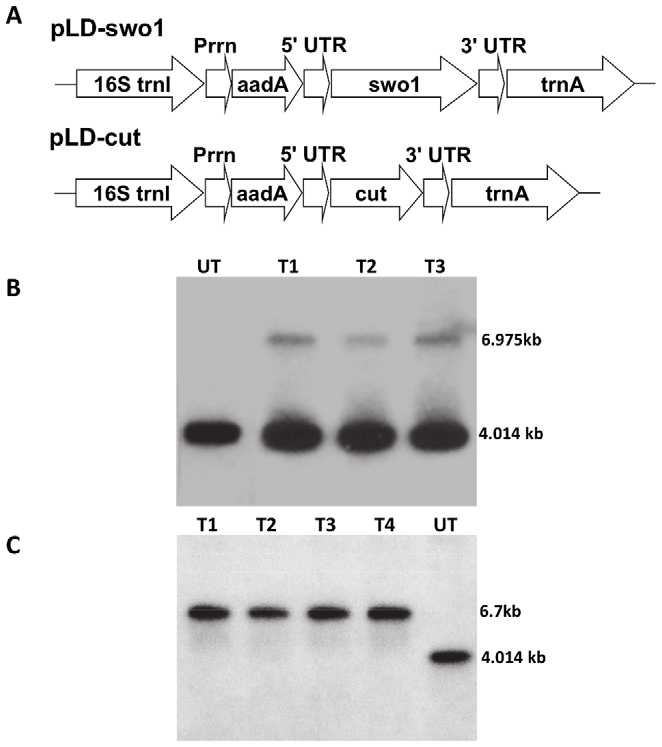
Evaluation of transgene integration and homoplasmy by Southern blot analysis. A, Schematic representation of the chloroplast transformation vectors pLD-swo1 and pLD-cut. Prrn, rRNA operon promoter; *aad*A, aminoglycoside 3′-adenylyltransferase gene; 5′ UTR, promoter and 5′ untranslated region of *psb*A gene; 3′ UTR, 3′ untranslated region of *psb*A gene. B, Southern blot analysis of independent cutinase transplastomic lines hybridized with chloroplast flanking sequence probe showing heteroplasmy (UT, untransformed; T1–T3, transplastomic lines). C, Southern blot analysis of independent swollenin transplastomic lines hybridized with chloroplast flanking sequence probe showing homoplasmy (UT, untransformed; T1–T4, transplastomic lines).

In pLD-swo1 and pLD-cut, *swo*1 and cutinase gene was regulated by the *psb*A promoter and 5′ UTR to accomplish higher expression levels (Figure 1A). The 3′ UTRs imparted stability to the transcript. Expression of the *aad*A gene under constitutive rRNA operon promoter and GGAG ribosome-binding site provided resistance to spectinomycin and was utilized for selection. The 16S-*trn*I/*trn*A sequences present in inverted repeat region of chloroplast genome were used to flank expression casettes in vectors for homologous recombination. The sequences in between *trn*I and *trn*A genes harbors a transcriptionally active region and have been used extensively for integration and hyper-expression of several transgenes [1], [60]–[65]. Transplastomic plants were recovered as explained earlier [54], [55]. PCR assay with two primer sets (3P/3M and 5P/2M) generated anticipated size fragments confirming integration of transgenes at precise location in the chloroplast genome (data not shown).

To further substantiate site-specific transgene integration into the chloroplast genome and to identify homoplasmic plants which harbored exclusively transformed chloroplast genomes, Southern blot analysis was carried out. Digestion of total plant DNA from transplastomic and untransformed shoots with the restriction enzyme *Sma*I followed by hybridization with a 0.81 kb flanking sequence probe generated a 4 kb fragment in untransformed or a 6.9 kb and 6.7 kb fragment for cutinase and swollenin transplastomic lines, respectively confirming proper transgene integration into the spacer region of *trn*I and *trn*A genes (Figure 1B and 1C). In addition, the 4 kb fragment was not detected in swollenin transplastomic lines confirming that homoplasmy has been attained (Figure 1C). However, only heteroplasmic plants were recovered in cutinase transplastomic lines (Figure 1B).

### Phenotypes of Transplastomic Lines and Chloroplast Ultrastructure

**Figure 2:**
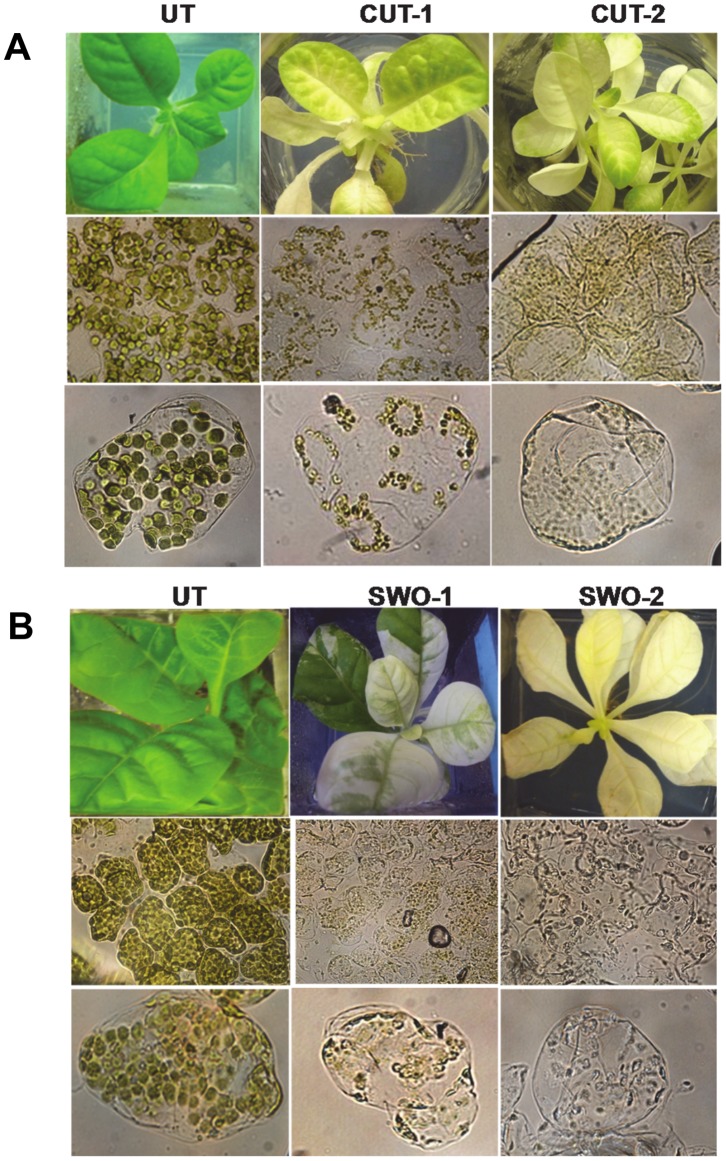
Microscopic evaluation of mesophyll cells. A, Untransformed and cutinase transplastomic lines (UT, untransformed; CUT-1 and CUT-2, cutinase transplastomic lines). Lower panel is an enlarged view. B, Untransformed and swollenin transplastomic lines (UT, untransformed; SWO1-1 and SWO1-2, swollenin transplastomic lines). Lower panel is an enlarged view. The chloroplasts were observed at 60X magnification under a Leica inverted microscope outfitted with a Leica camera.

Expression of cutinase and swollenin in tobacco chloroplasts resulted in drastic changes in plant phenotype and chloroplast structure. All the transplastomic lines showed pleiotropic effects including bleaching of leaves, slow growth and did not survive in soil. The leaves of cutinase and swollenin transplastomic plants appeared yellow/pale or white in color depending upon their level of expression (Figure 2A and 2B). However, under *in vitro* culture conditions, both transplastomic lines survived for a long time. The young swollenin plants (6- week old) showed unique mosaic-like leaf phenotype when compared to the normal green leaves of untransformed plants. Green portion of swollenin plants turned into pale or white after prolonged *in vitro* culture. The leaves of three-month old plantlets were completely pale in color and very thin when compared to untransformed leaves (Figure 2B). Beside the pale green leaves, the swollenin plants also showed weaker root system and slower growth. All heteroplasmic cutinase plants showed yellow or pale phenotype (Figure 2A).

In order to investigate the differences in chloroplast structure, leaf mesophyll cell chloroplasts were observed under the light microscope (60X). In both the transplastomic lines, the number and size of chloroplasts decreased significantly and showed fusion to form clusters (Figure 2A and 2B). Chloroplasts gradually decreased in size or were lysed. In mature transplastomic lines, almost all the internal structure of chloroplasts collapsed.

**Figure 3:**
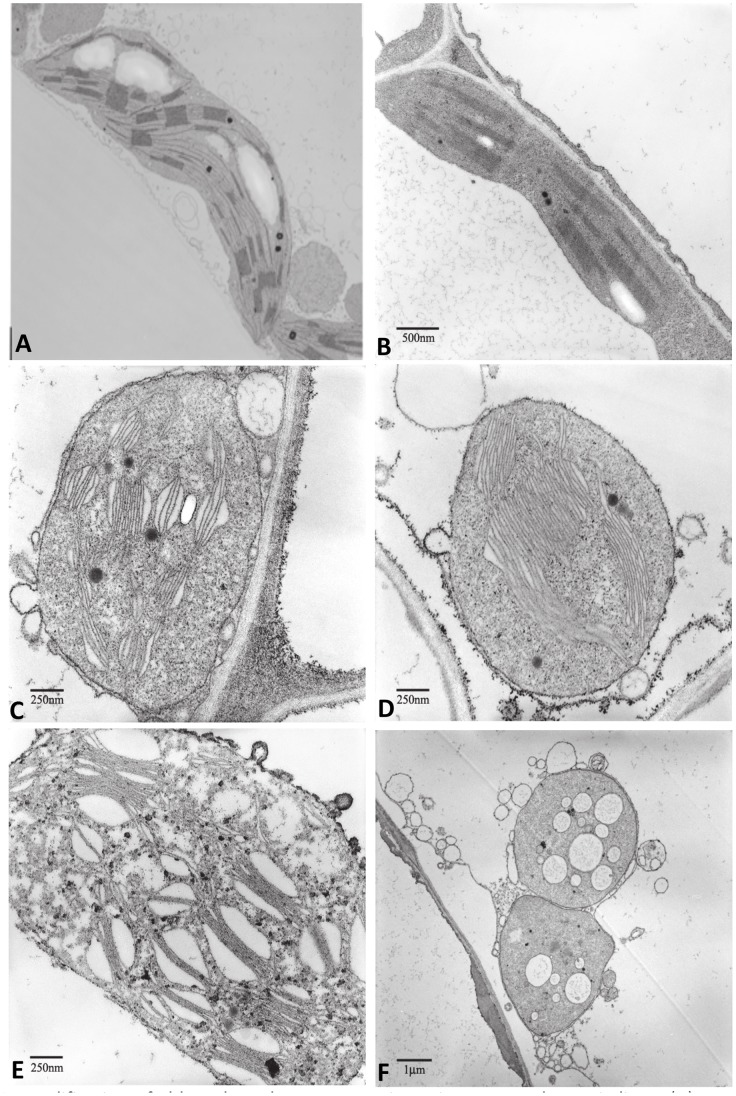
Modification of chloroplast ultrastructures in cutinase transplastomic lines. A, Transmission electron micrographs of chloroplasts from untransformed leaf tissue (10,000 X). B-F, Transmission electron micrographs of chloroplasts from cutinase transplastomic lines with (B) light green (20,000 X), (C-D) pale (31,500 X), (E) mosaic (31,500 X) and (F) pale leaf (8,000 X).

**Figure 4:**
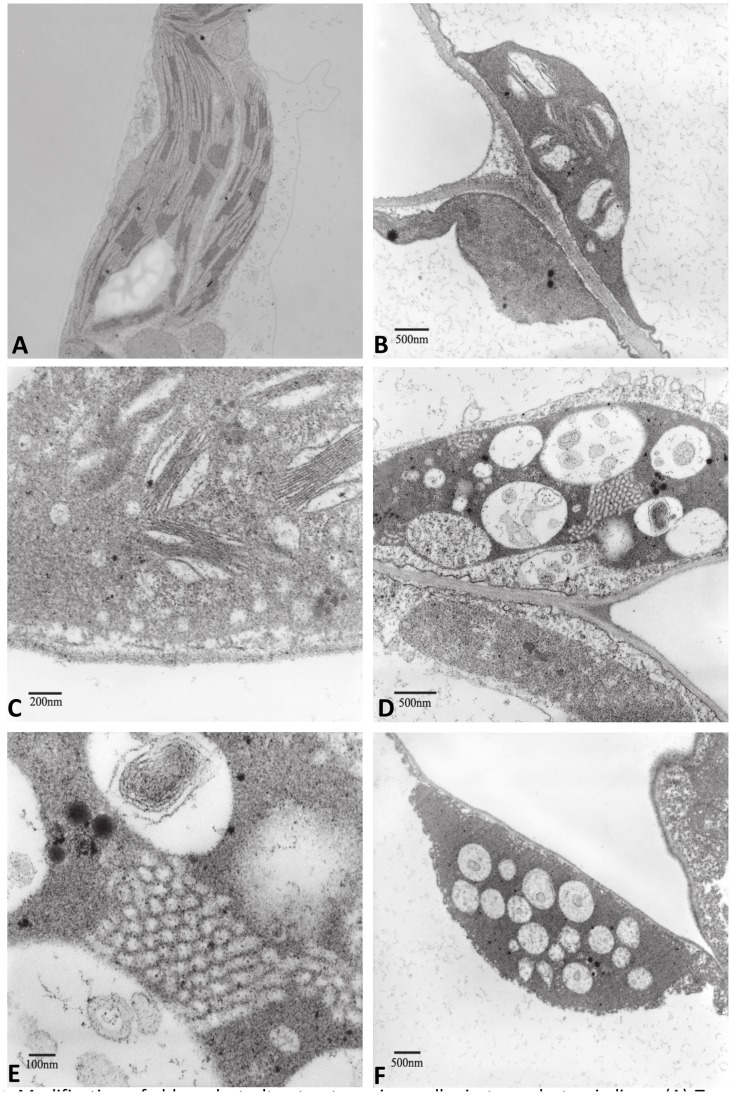
Modification of chloroplast ultrastructures in swollenin transplastomic lines. A, Transmission electron micrographs of chloroplasts from untransformed leaf tissue (10,000 X). B-F, Transmission electron micrographs of chloroplasts from swollenin transplastomic lines with (B) light green (16,000 X), (C) pale (40,000 X), (D) mosaic (20,000 X), (E) mosaic (63,000 X) and (F) pale leaf (12,500 X).

Chloroplasts of untransformed plants were lens-shaped having double membrane in the chloroplast envelope and a typical arrangement of parallel stromal thylakoids and compactly stacked granal thylakoids (Figure 3A and 4A). The ultrastructural organization of chloroplasts was significantly modified in cutinase (Figure 3B–3F) and swollenin (Figure 4B–4F) transplastomic plants and the extent of these changes increased from mosaic to white leaves. Ultrastructural analysis of chloroplasts from swollenin and cutinase transplastomic plants showed less thylakoid stacking and minimal thylakoid membranes. Both cutinase and swollenin expressing plants also showed swollen chloroplasts with disoriented thylakoid membranes. In pale green leaves, some chloroplasts showed round structure and lacked the lens shaped structure typically observed in mature chloroplasts. Some chloroplasts showed globular structures possibly generated by amalgamation of internal membranes. Also membrane fragments having scroll like structures without any stacking were observed. Complete disruption of the chloroplasts was also observed with accumulation of plastoglobuli representing the accumulation of lipids, released from the breakdown of the thylakoids. Some ultrastructural differences between chloroplasts of cutinase and swollenin transplastomic plants were observed. In swollenin transplastomic plants, many prothylakoid structures and prolammellar bodies were present without any grana. Prolamellar bodies are arranged in both tubular and in honey-comb like structure similar to those observed in etioplasts. Prolamellar bodies that occur in hexagonal arrangement are not connected to any other membrane structure. In both transplastomic plants outer chloroplast membrane appears to be perturbed.

### Effect of Swollenin Treatment on Mercerized Cotton Fiber

**Figure 5:**
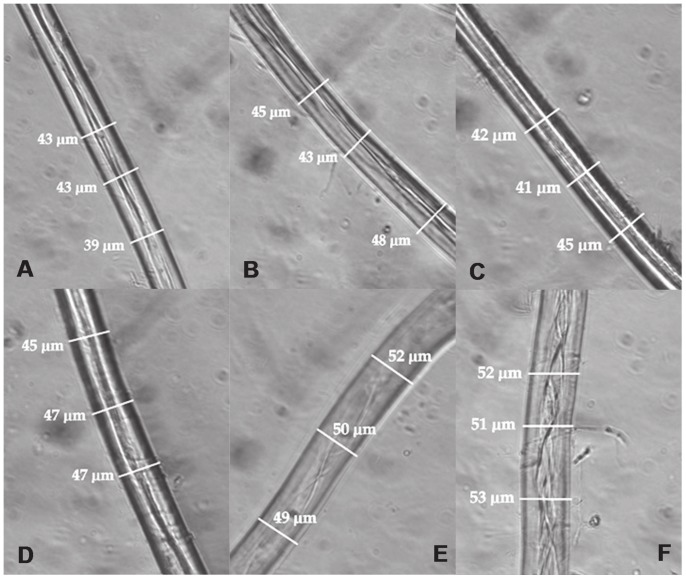
Phase contrast microscopic evaluation of cotton fiber treated with swollenin and cutinase. Average width of cotton fiber is given in brackets. A, Untransformed *E. coli* extract (41.6 µm). B, Purified cutinase (45.3 µm). C, Chloroplast cutinase (42.6 µm). D, Untransformed extract (46.3 µm). E, *E. coli* Swo1 (50.3 µm). F, Chloroplast Swo1 (52.0 µm).

Cotton fibers were visualized under light microscope after 4 hour incubation with protein extract from swollenin transplastomic lines. There was no increase in fiber diameter in samples treated with cpCutinase (Figure 5C) and the inner fiber arrangements remained nearly intact resulting in dark outer boundaries because enough optical light was not passing through. In the swollenin treated sample, the fibers were visibly swollen more and at many places the intertwined arrangements of inner fibers were thoroughly unwound and opened up (Figure 5E and 5F). Cotton fibers treated with swollenin showed fiber expansion after 8 hour incubation at 37°C or 50°C whereas untransformed plant extract and *E. coli* extract did not cause such expansion (Figure 5A and 5D). When mercerized cotton fibers were treated with purified cutinase or chloroplast-derived cutinase, no swelling was observed (Figure 5B and 5C). Furthermore, the chloroplast or E.coli derived swollenin didn’t show any detectable activity on various substrates including carboxymethyl cellulose, pNPG, microcrystalline cellulose, oat spelt xylan, and glucomannans (data not shown).

### Esterase and Lipolytic Activity of Chloroplast Expressed Cutinase

**Figure 6:**
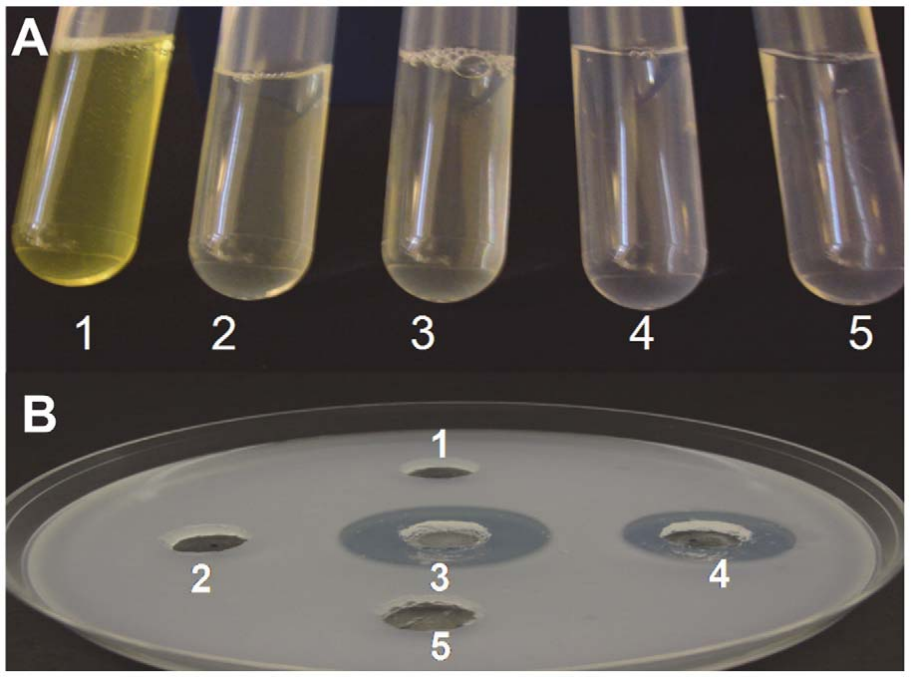
Lipolytic and esterase activity of cutinase. A, para-Nitrophenyl butyrate assay. 1, cp Cutinase; 2, cpSwollenin; 3, Untransformed; 4, *E. coli* swollenin; 5, Buffer alone; The para-nitrophenyl butyrate (pNPB, 0.01%) was incubated with different extracts for 30 min at 30°C and visualized. B, Tributyrin plate assay. 1, cpSwollenin; 2, Untransformed; 3, *E. coli* cutinase; 4, cpCutinase; 5, Buffer alone. Emulsified tributyrin (1%) in 2% agarose was used for plate assay. Different extracts were added to punch holes in plate followed by incubation at 37°C and visualized after 24 h.

Leaf extract from cutinase transplastomic lines and E.coli derived cutinase showed zone of clearance when incubated on tributyrin plate (Figure 6B). No zone of clearance was seen with buffer alone used as negative control. Also, the swollenin expressing plants and untransformed plants did not show any clearance zone (Figure 6B). The lipolytic activity of cpCutinase plants was measured spectrophotometrically with p-nitrophenyl butyrate as substrate. Cutinase expression and activity varied among *in vitro* plants due to their heteroplasmic nature. However, two lines (cut-1 and cut-2) showed hydrolysis activity of p-nitrophenyl butyrate substrate in the range of 9 to 15 units/mg tsp (Figure 6A). Untransformed plants, cpSwollenin, E.coli Swollenin and buffer alone did not show any hydrolysis of p-nitrophenyl butyrate substrate.

### Hydrolysis of 14C-DGDG by Purified Cutinase

**Figure 7:**
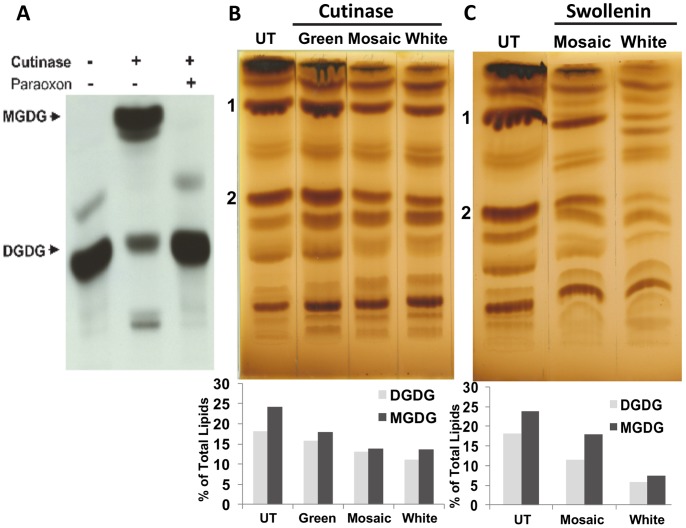
Autoradiogram and TLC showing lipid hydrolysis. A, Autoradiogram of TLC plate showing hydrolysis of the lipid DGDG by *F. solani pisi* cutinase is inhibited by active serine-directed inhibitor paraoxon. The ^14^C-DGDG was incubated with purified cutinase for 2 h at 37°C in the absence and presence of paraoxon (active serine-directed inhibitor). All reactions were extracted and resolved by silica-TLC. Positions of MGDG and DGDG corresponding to the migration of authentic unlabeled standards are shown. B, TLC of green, mosaic and white leaves from cutinase expressing transplastomic tobacco lines and percentage of MGDG and DGDG in total extracted lipids. C, TLC of mosaic and white leaves from swollenin expressing transplastomic tobacco lines and percentage of MGDG and DGDG in total extracted lipids. UT: leaf lipid extracted from untransformed plant. 1: MGDG. 2: DGDG. Lipid bands were visualized using iodine vapor and IDV values were calculated by densitometry.

Purified cutinase was assayed with ^14^C-DGDG as substrate followed by extraction of lipids and their separation on silica TLC plates. Cutinase effectively hydrolyzed ^14^C-DGDG (Figure 7A). To investigate the role of active serine residue of cutinase in the enzymatic hydrolysis of ^14^C-DGDG, the cutinase enzyme was treated with paraoxon, an active serine-directed inhibitor. The enzymatic hydrolysis vanished in the assay where the cutinase was treated with paraoxon (Figure 7A). Purified swollenin didn’t show any such hydrolysis.

### Reduction of Galactolipids in the Swollenin and Cutinase Transplastomic Lines

To examine the effect of expression of swollenin and cutinase in chloroplasts on plant lipids, we analyzed the total lipids composition extracted from untransformed and transplastomic plants. Figure 7 (B & C) shows the separation of total lipids achieved by thin-layer chromatography (TLC) and their visualization using iodine vapor. MGDG and DGDG were identified by comparison with standard lipids. Both MGDG and DGDG were strongly reduced in the cutinase and swollenin expressing plants. In the green leaf of cutinase plants, the amount of MGDG and DGDG was reduced to 17.94% and 15.61% respectively of total lipids confirming the loss of MGDG and DGDG, the two most important thylakoid lipids. In mosaic leaves, MGDG and DGDG were reduced by 43.1% and 28.8% respectively when compared with the untransformed leaves, whereas in white leaves the reduction was 47.7% and 39.7%. Levels of MGDG and DGDG were also reduced in mosaic leaves from swollenin plants by 25% and 37.3% when compared to the green leaves from untransformed plants. However in white leaves, both MGDG and DGDG contents drastically decreased by ∼68%. The ratio of MGDG/DGDG was ∼1.3, 1.1 and 1.3–1.5 in untransformed, cutinase and swollenin plants, respectively.

## Discussion

In this study, cutinase from *Fusarium solani* and swollenin from *Trichoderma reesei* were expressed in tobacco chloroplasts. Although, homoplasmy was achieved in swollenin transplastomic lines, all the cutinase lines were heteroplasmic. Expression of cutinase and swollenin in chloroplast caused drastic changes in plant phenotypes and chloroplasts structure. All the transplastomic plants had pleiotropic effects including bleaching of leaves, slow growth and did not survive in soil. The development of photosynthetic membranes is linked with the pigments synthesis and their precise integration into LHC complexes. Chlorophyll also has stabilizing effect on complexes and in the absence of chlorophyll the complex proteins become vulnerable for digestion by proteases [6]. All the transplastomic plants showed pale or white phenotype showing the lack of pigments. Because none of the genes involved in pigment biosynthesis are localized within the chloroplast genome, lack of pigment synthesis could not be the reason for observed results. Therefore, other possible mechanisms were investigated. Chloroplasts of both transplastomic lines showed degeneration symptoms such as reduction in chloroplast volume, unstacking and degradation of thylakoid membranes, immature thylakoid system, and increase in plastoglobuli.

The major photosynthetic light-harvesting pigment−protein complex LHCII is the most abundant membrane protein complex in thylakoid membranes. Both monogalactosyldiacylglycerol (MGDG) and digalactosyldiacylglycerol (DGDG) have definite functions in assembly of thylakoid membranes and are tied to light harvesting complex (LHC II). The stacked grana structure has ordered arrays of two dimensional crystals of LHCII whereas in unstacked membranes the arrays are absent [16]. The DGDG molecules allow the LHCII trimers to pack into crystalline structure and removal of DGDG makes the LHC II incapable of forming two- or three-dimensional crystals [15]. In this study, purified cutinase showed hydrolysis of ^14^C-DGDG by alpha galactosidase activity. Therefore, loss of grana stacking observed in cutinase expressing plants could be because of hydrolysis of DGDG by cutinase leading to the disruption of light harvesting complexes and unstacking of thylakoid membranes. Because, DGDG is hydrolyzed to MGDG by cutinase, levels of MGDG and DGDG were analyzed in leaf lipid extract. Surprisingly, reduction in both galactolipids as well as total lipids was observed in cutinase transplastomic lines and could be one of the causes for unique phenotype of cutinase lines. Tributyrin and para-nitrophenyl butyrate substrate assay showed that cutinase produced in transplastomic plants is biologically active and could hydrolyze other lipids. Cutinase lines also showed reduction in the number of chloroplasts. Altogether, hydrolysis of DGDG by cutinase, disorganised thylakoid membranes, esterase activity, less number of chloroplasts and decrease in the lipid content resulted in chlorotic phenotype of cutinase transplastomic plants.

Swollenin is an expansin type enzyme that has the potential to cause deagglomeration or amorphogenesis of cellulose fibers thereby facilitating entry of other cell wall degrading enzymes like cellulase, hemicellulases and pectinase into the complex and tightly bound, intertwined cellulose and hemicelluloses compounds for efficient hydrolysis [19], [66], [67]. Disruption of hydrogen bonds between cellulose fibers has been suggested as a possible mechanism of expansin function [20]. In our study, cotton fibers showed expansion without hydrolysis or release of reducing sugar ends when incubated in chloroplast-derived swollenin enzyme for 4 hours. Swollenin enzyme activity caused intertwined fibers to irreversibly open widely. Expansion of cotton fibers showed that the swollenin expressed in transplastomic plants is biologically active. In addition, chloroplast expressed swollenin as well as partially purified swollenin expressed in *E. coli* caused similar effects. In contrast, cutinase expressed in chloroplasts as well as purified cutinase that showed esterase and lipase activities did not cause swelling of cotton fibers. This observation is important to explain how swollenin caused tremendous structural damage to chloroplast double membrane and also complete disorganization of thylakoid membrane structure. We also investigated whether swollenin has other enzymatic activities including lipase or esterase using appropriate substrates. In all other enzymatic assays, swollenin did not show any of the tested enzymatic activities.

The swollenin protein is comprised of a cellulose binding domain (CBD) at N-terminus coupled to a homologous expansin domain by a linker region [19]. The CBD enables binding of swollenin protein to carbohydrates. The phenotype seen in swollenin transplastomic plants could be due to the carbohydrate binding property of swollenin resulting in altered carbohydrate metabolism. Thus far, hyperexpression of cellulolytic enzymes in chloroplasts rarely showed adverse phenotypic effects due to their compartmentalization inside the chloroplasts [1], [64], [68]. In fact, the transplastomic plants expressing beta-glucosidase showed enhanced biomass production when compared with untransformed plants [4]. Expression of an endoglucanase in apoplasts also did not show any phenotypic defects [69]. Overexpression of cell wall degrading enzymes in transplastomic plants showing phenotypic defects due to the capture or breakdown of carbohydrate metabolism intermediates has also been reported [3], [70]. Swollenin can also bind to starch and accelerate its hydrolysis into maltose resulting in altered phenotype due to modified starch metabolism. Further, swollenin transplastomic plants showed fewer chloroplasts and structural damage to thylakoid membranes as observed in light and electron micrographs. Arabidopsis plants harboring maltose excess 1 (mex1) mutation showed chlorotic phenotype due to excessive maltose accumulation [71]. The mex1 leaves contained less than 50% chloroplasts when compared to wild-type plants and showed less granal stacking with disrupted thylakoid membrane system. In addition, microarray analysis showed decrease in transcription of genes associated with proteins of light-harvesting complex [71]. The disruption of thylakoid membranes as observed under electron microscope in swollenin expressing plants (Figure 4) results in disassociation of galactolipids from light harvesting complexes. The disassociation of galactolipids is further enhanced by hydrogen bond disruption activity of swollenin. This makes galactolipids more vulnerable to degradation, thereby resulting in decreased galactolipids content in swollenin transplastomic plants (Figure 7C).

In conclusion, hydrolysis of DGDG substrate by cutinase is a novel function not reported or anticipated in published literature. Such hydrolysis led to disruption of pigment-protein complexes, unstacked granal thylakoids and disruption of chloroplast ultrastructure. On the other hand, swollenin demonstrated expansin function on cotton fibers and exhibited similar structural damage to chloroplasts followed by degradation of MGDG and DGDG by galactosidases and galactolipases. Both transplastomic lines showed fewer chloroplasts and decrease in galactolipid content. Swollenin transplastomic plants showed greater reduction of MGDG and DGDG than plants expressing cutinase. Cutinase transplastomic lines showed esterase and lipase activities whereas swollenin lines lacked such activities. Both swollenin and cutinase expressing plants showed similar phenotypic effects. To possibly lower or eliminate the phenotypic effects, the heterologous promoter or different regulatory elements could be used to generate swollenin or cutinase expressing plants with lower expression levels as an alternative to endogenous *psb*A promoter, which is used to achieve hyperexpression of transgenes [63]. The use of transplastomic leaves with lower levels of expression of swollenin should not be a problem in biomass hydrolysis for ethanol production as lesser concentration of swollenin has been shown to be adequate for its enhancing effect in maximum cellulosic substrate hydrolysis [66]. At the same time, more amount of cutinase transplastomic leaf material could be used to compensate for lower expression levels. The expression of cell wall degrading enzymes is very important to accomplish bioenergy goals along with the development of devoted bioenergy feedstocks [72]. In addition, dissection of cellulosic biomass pretreatment and enzymatic hydrolysis mechanisms should help in understanding complete digestion and release of sugars [73]. This study provides further understanding of the mechanism of both enzymes in membrane destabilization, in addition to identification of their novel activities and substrates.
